# Searchable attribute-based encryption scheme with attribute revocation in cloud storage

**DOI:** 10.1371/journal.pone.0183459

**Published:** 2017-08-31

**Authors:** Shangping Wang, Duqiao Zhao, Yaling Zhang

**Affiliations:** 1 School of Science, Xi’an University of Technology, Xi’an, Shaanxi, China; 2 School of Computer Science, Xi’an University of Technology, Xi’an, Shaanxi, China; Kaohsiung Medical University, TAIWAN

## Abstract

Attribute based encryption (ABE) is a good way to achieve flexible and secure access control to data, and attribute revocation is the extension of the attribute-based encryption, and the keyword search is an indispensable part for cloud storage. The combination of both has an important application in the cloud storage. In this paper, we construct a searchable attribute-based encryption scheme with attribute revocation in cloud storage, the keyword search in our scheme is attribute based with access control, when the search succeeds, the cloud server returns the corresponding cipher text to user and the user can decrypt the cipher text definitely. Besides, our scheme supports multiple keywords search, which makes the scheme more practical. Under the assumption of decisional bilinear Diffie-Hellman exponent (*q*-BDHE) and decisional Diffie-Hellman (DDH) in the selective security model, we prove that our scheme is secure.

## Introduction

In 2005, Waters et al.[1] came up with the concept of ABE(Attribute-Based Encryption) which was much more flexible than traditional public-key encryption. With the development and deepening of ABE, the attribute revocation of ABE is concerned by more and more people. The efficient attributes revocation scheme is an integral part of ABE scheme, which is one of the difficulties for the application of ABE, and the study of ABE is inseparable from the attribute revocation scheme research.

P. Traynor et al.[2] put forward a scheme which achieved the update of secret key in 2006. However, it needed that the user must kept close contact with attribute authority to get the secret key. Thereafter, Kumar et al.[3] presented a scheme with revocation of ABE, and it expanded from the IBE which they proposed before. All of these articles demand that users need to access the attribute authority for key reissuing at regular intervals.

In 2008, Jiang et al.[4] gave a scheme that solved the key misused problem of users. However, in this scheme, the third party should be included in each decryption key of users, and made it was unrealistic. After that, Kim et al.[5] inserted the users’ information in the secret key of attribute by using the black box model and sent it to the user, which was more efficient to guarantee the security of the system.

Attrapadung et al.[6] put forward the two revocation models, they are direct revocation model and indirect revocation model. The direct revocation model is specified the revocation list by sender, and the indirect revocation model updates the secret key periodically by the key center. In [7] [8], the authors gave some ABE instances. However, in the above schemes, they do not relate to the keyword search issue, which makes users can not effectively search for files.

To overcome this problem, Boneth et al. [9] proposed a single keyword search scheme, namely the user can only search a single keyword. In this scheme, the data owner extracted the keywords from the file before encrypted, and used the public key to encrypt the keywords. After that, the data owner sent the file and the index of the keywords to the cloud server. The user could generate the search token about the keywords which he wanted to search and sent it to the cloud server. The cloud server used the matching algorithm to find out the cipher text and returned it if the match was successful.

Searchable encryption has many practical applications. In 2011, Kerschbaum et al.[10] proposed a secure conjunctive keyword searches for unstructured text scheme, and the scheme was proved secure in the random oracle model. At the same year, Cao et al.[11] and Chuanh et al.[12] gave schemes that the multi-keyword search over encrypted data.

In 2014, Han et al. [13] proposed an attribute based encryption (ABE) searchable scheme, in which used the homomorphic encryption technology. Sahai et al. [14] gave a outsourcing technique based on the scheme of Gentry et al.[15]. After that, Liang K et al. [16] proposed a searchable ABE mechanism with efficient and secure in cloud storage. This model can be applied to real life, such as the safety of electric power system. And the scheme is secure in the random oracle model. Later, Li et al. [17] proposed a searchable ABE scheme with attribute revocation in cloud storage.

Willy Susilo et al.[18] proposed a searchable scheme, and it supported multiple keywords search. At the same time, Li J et al.[19] made a searchable CP-ABE with revocation. In this scheme, the receivers could not steal any information from the cipher because of the access structures were partially hidden, which made the scheme more secure.

In 2016, Wen et al. [20] proposed a verifiable attribute-based keyword search scheme with fine-grained owner-enforced search authorization in the cloud. This scheme supports user revocation. Besides, it allows data owners encrypt the data and outsource to the cloud server. In the same year, Yang et al. [21] proposed a conjunctive keyword search scheme with designated tester. User can search within a specified time if he is authorized, and it is proved secure in the standard model. In 2017, Jiang et al. [22] proposed a keyword search scheme with efficiency and verification in cloud data, and it allows multi-keyword search. Finally, they gave the security analysis in the scheme. Later, Poon et al.[23] constructed a conjunctive keyword search scheme. This scheme allows phrase search, and has smaller storage cost.

### Our contribution

In 2012, Qiang Li et al.[24] put forward a scheme with fine-grained attribute revocation. However, the scheme only achieves the attribute revocation, the keyword search is not involved, this problem may lead to the problem that system users cannot effectively download cipher text which they interested from the cloud server.

In this paper, we propose a keyword search attribute based encryption scheme with attribute revocation. The new scheme supports not only the attribute revocation but also keyword search. When a user wants to search the file which he interests, he sends the search token to the cloud server, and the cloud server runs the test algorithm. If the test is successful, it returns the file. In this way, the user can download the file which he interests and save the storage space at the same time. Finally, under the assumption of *q*-BDHE and DDH in the selective security model, we prove that our scheme is secure.

## Preliminaries

A linear secret sharing scheme can be used to represent an access control policy (*M*, *ρ*), which *M* is an *l*×*k* matrix, and *S* = {*att*_1_, …, *att*_n_} be an attribute set, and for *i* ∈ [1,*l*], *ρ*(*i*) → *S* is a mapping function, and *ρ*(*i*) maps a row into the attribute.

### Linear Secret-Sharing Scheme (LSSS) [25]

A linear secret sharing scheme includes two algorithms:

Share: In this step, it is dispersing the secret value *s* to attributes specified by *ρ* as follows: by selecting $v_{2},\ldots ,v_{k}\overset{R}{\to }Z_{p}$,setting $\vec{V}=(s,v_{2},\ldots ,v_{k})$ and computing $\lambda_{i}=M_{i}\cdot \vec{V}$ where *M*_*i*_ is the *i*th row of *M*,it assigns secrets share *λ*_*i*_ to the attribute *ρ*(*i*).

Combine: In this step, it is used to collect the secret value from secret shares which related to the attributes as follows: selecting subset *I* = {*i*: *ρ*(*i*) ∈ *S*} the attribute set {*ρ*(*i*) | *i* ∈ *I*} satisfies access control strategy (*M*, *ρ*), and computing coefficients *k*_i_, *i* ∈ *I* such that ∑_*i*∈*I*_ *k*_*i*_*M*_*i*_ = (1,0,…, 0), then we will obtain that ∑_*i*∈*I*_ *k*_*i*_*λ*_*i*_ = *s*.

### Decisional *q*-BDHE assumption [24]

The definition of the decisional *q*-BDHE exponent assumption in our article as follows:

Choose a group *G*_1_ of prime order *p*, let *g* be a generator of *G*_1_, and define *e*: *G*_1_ × *G*_1_ → *G*_2_, the adversary is given a vector

$$(g,g^{s},g^{a},g_{}^{a^{2}},\ldots ,g_{}^{a^{q}},g_{{}_{}}^{a^{q+2}},\ldots ,g_{}^{a^{2q}})\in G_{1}^{2q+1}$$

We say that the Decision *q*-BDHE assumption holds in *G*_1_ if no polynomial-time algorithm has a non-negligible advantage to distinguish $e{\left(g,g\right)}^{sa^{q+1}}$ and a random element in *G*_2_.

### Zero Inner-product [24]

The ID represents the identity of user which associated with user’s private key. Define a vector **X** = (*x*_1_,…,*x*_*n*_)^T^ such that *x*_*i*_ = *ID*^*i*-1^, *i* ∈ [1, *n*]. To encrypt with a revoked user set *R* = {*ID*_1_,⋯, *ID*_*q*_}, one defines as **Y** = (*y*_1_,…, *y*_*n*_)^T^, the coefficient vector of *P*_*R*_[*Z*] from

$$P_{R}[Z]=\sum_{i=1}^{q+1}y_{i}Z^{i-1}=\prod_{ID_{j}\in R}\left(Z-ID_{j}\right)$$

where, if *q* + 1 < *n*, the coordinates *y*_*q*+2_,⋯,*y*_*n*_ are set to 0. By doing so, we note that *P*_*R*_[*ID*] = <**X**, **Y**> = 0 iff *ID* ∈ *R*.

For example, if the user *ID*_1_ in the revoked user set *R* = {*ID*_1_, *ID*_3_}, we have that $P_{R}[ID_{1}]=<X,Y>=\prod_{ID_{j}\in R}\left(ID_{1}-ID_{j}\right)=0$.

### Decisional DDH assumption [10]

Let *G*_1_ is a group which prime order is *p*, let *g* be a generator of *G*_1_, and give a tuple (*g*, *g*^*a*^, *g*^*b*^) where $a,b\overset{R}{\in }Z_{p}$, we say that the decisional DDH assumption holds if no polynomial time algorithm has a non-negligible advantage to distinguish that *Z* equals *g*^*ab*^ or to a random element of *G*_1_.

### Algorithm model and security model

#### Algorithm model

**Fig 1:**
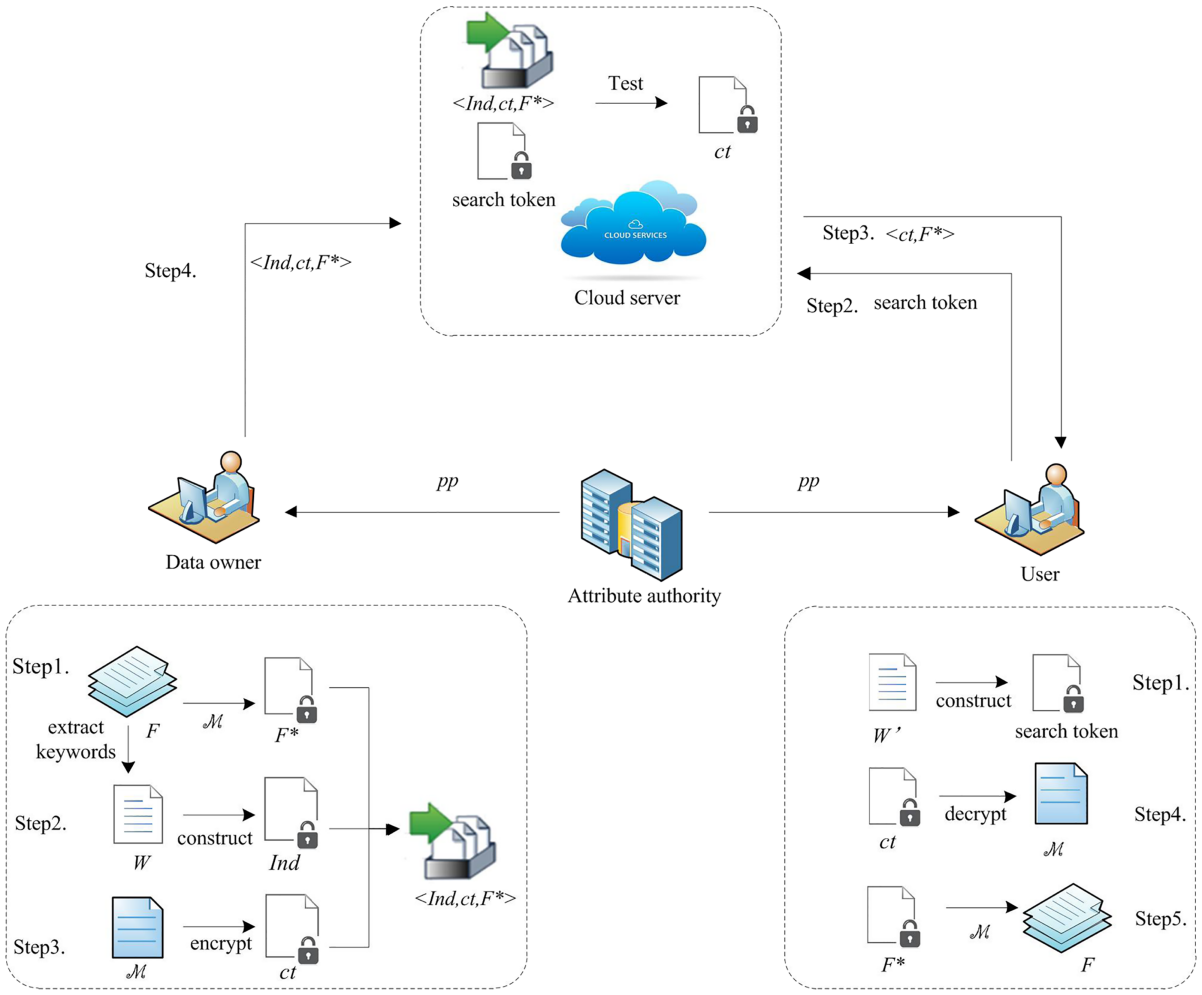
System model of our scheme

Denote *U* = {*ID*_1_,⋯, *ID*_*Q*_} to be the universe of all the users, we consider a scheme that searchable attribute-based encryption scheme with attribute revocation in cloud storage, as described in Fig 1. There are seven algorithms in our scheme:

**Setup** (*λ*) → *msk*, *pp*: This algorithm is executed by attribute authority. It inputs a security parameter *λ* and outputs the master secret key *msk* and public parameter *pp*.

**KeyGen** (*ID*, (*M*, *ρ*), *pp*, *msk*) → *sk*, *τ*:This algorithm is executed by attribute authority. It inputs a user’s identity *ID* ∈ *U*, an access structure (*M*, *ρ*), public parameter *pp*, the *msk* and outputs the secret key *sk* and the part of search token *τ*.

**Encryption** (*pp*, *ω*, *R*_*θ*_, *m*) → *ct*: This algorithm is executed by data owner. It inputs public parameter *pp*, the attribute set *ω*, a revocation list *R*_*θ*_ ⊆ *U* which attribute *θ* ∈ *ω*,a message *m* and outputs a cipher text *ct*.

**Index** (*pp*, *ω*, *R*_*θ*_, *W*) → *Ind*: This algorithm is executed by data owner. It inputs public parameter *pp*, the attribute set *ω*,a revocation list *R*_*θ*_ ⊆ *U* which attribute *θ* ∈ *ω*,the keywords set from the uploaded files *W* and outputs keywords index *Ind*.

**Trapdoor** (*pp*, *W*′, *τ*) →τ*:This algorithm is executed by user. It inputs the public parameter *pp* and the keywords set *W*′, and outputs the new token τ*.

**Test** (τ*, *Ind*) → 1 *or* 0:This algorithm is executed by cloud storage server. It inputs the search token τ*and keywords index *Ind* and outputs 1 or 0.

**Decryption** (*pp*, *ID*, *sk*, *R*_*θ*_, *ct*) → *m*: This algorithm is executed by user. It inputs public parameter *pp*, the user secret key *sk* of user *ID* ∈ *U*, a revocation list *R*_*θ*_ ⊆ *U* of attribute *θ* ∈ *ω*, a cipher text *ct*. And the user *ID* has the attribute set *ω*′ as: if *ID* ∈ *R*_*θ*_, let *ω*′ = *ω* − {*θ*};otherwise, *ω*′ = *ω*. It computes the message *m* if and only if the attribute set *ω*′ satisfies the access structure. And the user can decrypt the file with *m*.

Finally, the system model of our scheme is shown in Fig 1.

### Security model

#### (1) Selective security model of attribute revocation

**Init**. The adversary A chooses the attribute set ω* and a revocation list $R_{\theta }^{*}(\theta \in \omega^{*})$.

**Setup**. The simulator operates this algorithm to get the public parameter *pp* and sends it to the adversary.

**Phase 1**. The adversary queries the simulator for user private key *sk* which corresponds to the access structure (*M*, *ρ)*, such that ω*′will not meet the access structure (*M*, *ρ)*.

**Challenge**. The simulator receives two messages *m*_0_ and *m*_1_ from adversary, and chooses a random bit *b* ∈ {0, 1} to encrypt *m*_*b*_, and computes challenge cipher text ct* with the attribute set ω* and the attribute revocation list $R_{\theta }^{*}$.

**Phase 2**. Same as **Phase 1**.

**Guess**. The adversary gives a guess *b*′ of *b*, and the advantage of the adversary in this game is defined as $\left|\text{Pr}[b^{\prime }=b]-\frac{1}{2}\right|$.

**Definition1**. The game model of this paper is to be safe if there no polynomial time adversaries have a non-negligible advantage in the above game.

#### (2) Indistinguishability against chosen keyword attack (IND-CKA) model

**Init**. The adversary A selects a attribute set ω* and a user revocation list $R_{\theta }^{*}$ of *θ* ∈ ω*. Then B runs the algorithm to generate the public parameter *pp* and sends it to adversary A.

**Phase 1**. The adversary queries the challenger as follows:

1. The index of keywords {*w*_1_, *w*_2_,…, *w*_*N*_}.
2. The search token of $\left\{w_{j_{1}},w_{j_{2}},\ldots ,w_{j_{N_{1}}}\right\}$, and $1\leq j_{1},\ldots ,j_{N_{1}}\leq N$.

**Challenge**. The challenger receives two different keywords $w_{0}^{*}$ and $w_{1}^{*}$ from the adversary. We require that the keywords $w_{0}^{*}$ and $w_{1}^{*}$ satisfies that $\forall j,w_{j}\neq w_{0}^{*}\wedge w_{j}\neq w_{1}^{*}$.

The challenger chooses a random keyword $w_{b}^{*}$, *b* ∈ {0,1}, and give the index of keywords $w_{b}^{*}$ to adversary.

**Phase 2**. Same as **Phase 1**.

**Guess**. The adversary gives a guess *b*′ of *b*, and the advantage of any adversary in this game is defined as $\left|\text{Pr}[b^{\prime }=b]-\frac{1}{2}\right|$.

**Definition 2**. We say a searchable encryption article with multiple keywords is secure based on the game IND-CKA, if the advantage of the adversary is negligible in the above game.

### Implement of the algorithm

Our construction is based on the Qiang Li et al.[24], and we combine the keyword search with attribute revocation in our new scheme. User constructs the search token when he wants to search files. If the search is successful and the set of attribute satisfies the access structure, it outputs 1 in the algorithm of Test, then cloud server returns the cipher text. Our scheme adds access control in search, the user can download the files which he interests and can decrypt in this way, and save the space. We construct our scheme as follows:

**Setup** (*λ*) → *msk*, *pp*: Give that the *G*_1_ and *G*_2_ are two groups of prime order *p*, the binary size of *p* is *λ*,let *g* be a generator of *G*_1_. Define that *e*: *G*_1_× *G*_1_ →*G*_2_. In this paper, we suppose the maximum number of attribute is *m* when encryption, and *n* represents the maximum number of revoked user set in the revocation list. Then randomly choose *α*, *β*, *δ* ∈ *Z*_*p*_, $A={\left(\alpha_{1},\alpha_{2},\ldots ,\alpha_{n}\right)}^{\text{T}}\in Z_{p}^{n}$, set $H={\left(h_{1},h_{2},\ldots ,h_{n}\right)}^{\text{T}}={\left(g^{\alpha_{1}},g^{\alpha_{2}},\ldots ,g^{\alpha_{n}}\right)}^{\text{T}}$ and randomly choose {*k*_0,*i*_, *k*_1,*i*_ ∈ *G*_1_|_*i* = 1,…,*m*_},let $K_{0}(x)=\prod_{i=1}^{m}k_{0,i}^{\left(x^{i}\right)},K_{1}(x)=\prod_{i=1}^{m}k_{1,i}^{\left(x^{i}\right)}$. Then randomly choose that {*t*_0,*i*_, *t*_1,*i*_ ∈ *G*_1_|_*i* = 1,…,*m*_},and then define two functions *T*_*f*_(*x*): *Z*_*p*_ → *G*_1_,$T_{f}(x)=\prod_{i=1}^{m}t_{f,i}^{\left(x^{i}\right)}$ where *f* = {0, 1}. Let hash *H* be *H*:{0, 1}* → *G*_1_, then the master key *msk* and public parameter *pp* are:

$$msk=<\alpha ,\alpha_{1},\beta ,{\left\{k_{0,i},k_{1,i},t_{0,i},t_{1,i}\right\}}_{i=1,\ldots ,m}>$$

$$pp=<g,e{\left(g,g\right)}^{\alpha },H={\left(h_{1},h_{2},\ldots ,h_{n}\right)}^{\text{T}},g^{\beta },\delta ,H,K_{0}(x),K_{1}(x)>$$

**KeyGen** (*ID*, (*M*, *ρ*), *pp*, *msk*) → *sk*, *τ* : Let *M* be an *l* × *k* matrix corresponding to access policy (*M*, *ρ*). Define a vector **X** = (*x*_1_,…,*x*_*n*_)^T^ such that *x*_*i*_ = *ID*^*i*−1^, *i* ∈ [1, *n*]. Randomly choose *r*, {*z*_*i*,0_, *z*_*i*,1_}_i∈[2,…*k*]_ ∈ *Z*_*p*_, define a vector **v**_0_ = (*α + rα*_1_, *z*_2,0_,…, *z*_*k*,0_)^T^, **v**_1_ = (*α*, *z*_2,1_,…, *z*_*k*,1_)^T^. For *i* = 1 to *l*, and compute that *λ*_*i*,0_ = *M*_*i*_·**v**_**0**_ and *λ*_*i*,1_ = *M*_*i*_·**v**_**1**_. Randomly choose {*r*_*i*,0_, *r*_*i*,1_}_i∈[1,…*l*]_ ∈ *Z*_*p*_, and set the private key as

$$sk=<D_{1,0},D_{1,1},D_{2,0},D_{2,1},D_{3},K_{X}>$$

where

$$D_{1,0}={\left\{D_{1,0}^{\left(i\right)}=g^{\lambda_{i,0}}T_{0}{\left(\rho (i)\right)}^{r_{i,0}}\right\}}_{i\in [1,\ldots ,l]}$$

$$D_{2,0}={\left\{D_{2,0}^{\left(i\right)}=g^{r_{i,0}}\right\}}_{i\in [1,\ldots ,l]}$$

$$D_{1,1}={\left\{D_{1,1}^{\left(i\right)}=g^{\lambda_{i,1}}T_{1}{\left(\rho (i)\right)}^{r_{i,1}}\right\}}_{i\in [1,\ldots ,l]}$$

$$D_{2,1}={\left\{D_{2,1}^{\left(i\right)}=g^{r_{i,1}}\right\}}_{i\in [1,\ldots ,l]}$$

$$D_{3}=g^{r},K_{X}={\left\{K_{i}={\left(h_{1}^{-\frac{x_{i}}{x_{1}}}\cdot h_{i}\right)}^{r}\right\}}_{i\in [2,\ldots ,n]}$$

Then calculate that $K_{X}=(K_{2},\ldots ,K_{n})=g^{r\cdot M_{X}^{\text{T}}A}$, where *M*_**X**_ ∈ (*Z_p_*)^*n*×(*n*−1)^ is defined by $M_{X}=\left(\begin{array}{c} -\frac{x_{2}}{x_{1}}-\frac{x_{3}}{x_{1}}\cdots -\frac{x_{n}}{x_{1}}\\ I_{n-1} \end{array}\right)$.

Randomly choose $\{v_{2},\ldots ,v_{k}\}\in Z_{p}^{k-1}$ and set $v={\left(\beta ,v_{2},\ldots ,v_{k}\right)}^{\text{T}}\in Z_{p}^{k}$. For *i* = 1 to *l*, compute *λ*_*i*_ = *M*_*i*_·**v**. Randomly choose *ξ*_*i*_ ∈ *Z*_*p*_, then denote that

$$\tau =<\tau_{1},\tau_{2,0},\tau_{2,1}>$$

where

$$\begin{array}{lll} \tau_{1} & = & {\left\{\tau_{1,i}=g^{\lambda_{i}}\right\}}_{i=1,\ldots l}\\ \tau_{2,0} & = & {\left\{\tau_{2,0}^{\rho (i)}=K_{0}^{\xi_{i}}(\rho (i))\right\}}_{i=1,\ldots l}\\ \tau_{2,1} & = & {\left\{\tau_{2,1}^{\rho (i)}=K_{1}^{\xi_{i}}(\rho (i))\right\}}_{i=1,\ldots l} \end{array}$$

then send *sk* and *τ* to the user.

**Encryption** (*pp*, *ω*, *R*_*θ*_, *m*) → *ct*: Suppose that a message *m* is encrypted with a set of attribute *ω* and a revocation list *R*_*θ*_ ⊆ *U* which attribute *θ* ∈ *ω*. Define a vector **Y** = (*y*_1_,…, *y*_*n*_)^T^ as the coefficient vector of $P_{R_{\theta }}[Z]$, and randomly choose *s* ∈ *Z*_*p*_ then output

$$ct=\langle C,C_{1},C_{2,0},C_{2,1},C_{3}\rangle$$

where

$$C=m\cdot e{\left(g,g\right)}_{}^{\alpha s},C_{1}=g^{s}$$

$$C_{2,0}={\left\{C_{{}_{2,0}}^{\left(x\right)}=T_{0}{\left(x\right)}^{s}\right\}}_{x\in \omega },C_{2,1}={\left\{C_{{}_{2,1}}^{\left(x\right)}=T_{1}{\left(x\right)}^{s}\right\}}_{x\in \omega -\left\{\theta \right\}}$$

$$C_{3}={\left(h_{1}^{y_{1}}\cdots h_{n}^{y_{n}}\right)}^{s}$$

**Index** (*pp*, *ω*, *R*_*θ*_, *W*) → *Ind*: A revocation list *R*_*θ*_ ⊆ *U* which attribute *θ* ∈ *ω*. Data owner encrypts the file *F* which is firstly encrypted by a symmetric encryption algorithm and gets cipher text F*, and suppose that the symmetric encryption key is *m*. The set of keywords *W* = {*w*_1_, *w*_2_,…, *w*_*N*_} is extracted from the *F*, and randomly choose *t* ∈ *Z*_*p*_,and output the keywords index

$$Ind=<I_{0},I_{1,j},I_{2,0},I_{2,1}>$$

where

$$I_{0}=g^{t}$$

$$I_{1,j}=g^{\beta }\cdot H{\left(w_{j}\right)}^{\delta },j\in [1,N]$$

$$I_{2,0}={\left\{I_{2,0}^{\left(x\right)}=K_{0}^{t}(x)\right\}}_{x\in \omega },\;I_{2,1}={\left\{I_{2,1}^{\left(x\right)}=K_{1}^{t}(x)\right\}}_{x\in \omega -\theta }$$

and send <*Ind*, *ct*, F*> to the cloud server.

**Trapdoor** (*pp*, *W*′, *τ*) →τ*: The user constructs the search token τ* according to the keywords $W^{\prime }=\{w_{j_{1}},w_{j_{2}},\ldots ,w_{j_{N_{1}}}\},(1\leq j_{1},\ldots ,j_{N_{1}}\leq N)$ which he interests as

$$\tau_{3}={\left\{\tau_{1,j_{q}}=g^{\beta }\cdot H{\left(w_{j_{q}}\right)}^{\delta }\right\}}_{q=1,\ldots ,N_{1},j_{q}=1,\ldots ,N}$$

and sends search token τ* = < τ_1_, *τ*_2,0_, *τ*_2,1_, *τ*_3_> and his *ID* to the cloud server.

**Test** (τ*, *Ind*) → 1 *or* 0: The cloud server receives the search token from the user. First, the cloud server judges that whether the *ID* of user is in the revocation list *R*_*θ*_. If *ID* ∈ *R*_*θ*_, let *ω*′ = *ω* − {*θ*};otherwise, *ω*′ = *ω*. If the set *ω*′ satisfies the access structure (*M*, *ρ*), then there exists a set of constants {*μ*_*i*_ ∈ Z_*p*_}_*i*∈*I*_, such that $\sum_{i\in I}\mu_{i}\cdot M_{i}=(1,0,\ldots ,0)$.

(1) When *ID* ∉ *R*_*θ*_, cloud server selects *N*_1_ keywords index from the *Ind*, we denote the result of selecting as $\left\{I_{1,O_{1}},I_{1,O_{2}},\ldots I_{1,O_{N_{1}}}\right\}$,where $1\leq O_{1},\ldots ,O_{N_{1}}\leq N$. Then cloud server tests the selected index set $\left\{I_{1,O_{1}},I_{1,O_{2}},\ldots I_{1,O_{N_{1}}}\right\}$ with the search token τ* = < *τ*_1_, *τ*_2,0_, *τ*_2,1_, *τ*_3_> with the following equation

$$\prod_{q=1}^{N_{1}}e(I_{1},\tau_{1,j_{q}})\overset{?}{=}\prod_{\sigma =1}^{N_{1}}e(I_{1},I_{1,O_{\sigma }})$$

If the equation holds, it turns to next step; otherwise, it outputs 0.

$$\frac{e{\left(I_{0},\prod_{i\in I}(\tau_{1,i}\cdot \tau_{2,0}^{\rho (i)}\right)}^{\mu_{i}})}{e({\prod_{i\in I}\left(I_{2,0}^{\rho (i)}\right)}^{\mu_{i}},g)}\overset{?}{=}e(I_{0},I_{1})$$

If the equations all hold, it returns the corresponding cipher text <*ct*, F*> to the user, and user can decrypt. Otherwise, it outputs 0.

(2) When *ID* ∈ *R*_*θ*_, cloud server selects *N*_1_ keywords index from the *Ind*, we denote the result of selecting is $\left\{I_{1,O_{1}},I_{1,O_{2}},\ldots I_{1,O_{N_{1}}}\right\}$,where $1\leq O_{1},\ldots ,O_{N_{1}}\leq N$. Then cloud server tests the selected index set $\left\{I_{1,O_{1}},I_{1,O_{2}},\ldots I_{1,O_{N_{1}}}\right\}$ with the search token τ* = < *τ*_1_, *τ*_2,0_, *τ*_2,1_, *τ*_3_> with the following equation

$$\prod_{q=1}^{N_{1}}e(I_{1},\tau_{1,j_{q}})\overset{?}{=}\prod_{\sigma =1}^{N_{1}}e(I_{1},I_{1,O_{\sigma }})$$

If the equation holds, it turns to next step; otherwise, it outputs 0.

$$\frac{e{\left(I_{0},\prod_{i\in I}(\tau_{1,i}\cdot \tau_{2,1}^{\rho (i)}\right)}^{\mu_{i}})}{e({\prod_{i\in I}\left(I_{2,1}^{\rho (i)}\right)}^{\mu_{i}},g)}\overset{?}{=}e(I_{0},I_{1})$$

If the equations all hold, it returns the corresponding cipher text <*ct*, F*> to the user, and user can decrypt. Otherwise, it outputs 0.

**Decryption** (*pp*, *ID*, *sk*, *R*_*θ*_, *ct*) → *m*: User can decrypt according to the returned cipher text. If *ID* ∈ *R*_*θ*_, *ω*′ = *ω* − {*θ*};otherwise, *ω*′ = *ω*, and then:

(1) When *ID* ∈ *R*_*θ*_, let *I* = {*i*: *ρ*(*i*) ∈ *ω*′}, and there exists a set of constants {*μ*_*i*_ ∈ Z_*p*_}_*i*∈*I*_, such that ∑_*i*∈*I*_ *μ*_*i*_ · *M*_*i*_ = (1,0,…, 0),then ∑_*i*∈*I*_ *μ*_*i*_*λ*_*i*,1_ = *α*. It calculates

$$\varphi ={\prod_{i\in I}\left(\frac{e(C_{1},D_{1,1}^{\left(i\right)})}{e(C_{2,1}^{\rho (i)}),D_{2,1}^{\left(i\right)}}\right)}^{\mu_{i}}=e{\left(g,g\right)}^{s\alpha }$$

and *m* = *C* / *φ*, user can decrypt F* to get *F* with *m*.

(2) When *ID* ∉ *R*_*θ*_, calculate

$$K_{X}=\prod_{i=2}^{n}K_{i}^{y_{i}}={\left(h_{1}^{-\frac{<X,Y>}{x_{1}}}\prod_{i=1}^{n}h_{i}^{y_{i}}\right)}^{r}$$

so that when <**X**, **Y**> ≠ 0, and then calculate

$$\phi ={\left(\frac{e(K,C_{1})}{e(C_{3},D_{3})}\right)}^{-\frac{x_{1}}{<X,Y>}}=e{\left(g,g\right)}^{rs\alpha_{1}}$$

Let *I* = {*i*: *ρ*(*i*) ∈ *ω*′}, and there exists a set of constants {*μ*_*i*_ ∈ Z_*p*_}_*i*∈*I*_, such that ∑_*i*∈*I*_ *μ*_*i*_ · *M*_*i*_ = (1,0,…, 0),then ∑_*i*∈*I*_ *μλ*_*i*,*0*_ = *α*+ *rα*_1_. Thus we have

$$\gamma ={\prod_{i\in I}\left(\frac{e(C_{1},D_{1,0}^{\left(i\right)})}{e(C_{2,0}^{\rho (i)},D_{2,0}^{\left(i\right)})}\right)}^{\mu_{i}}=e{\left(g,g\right)}^{s\cdot (\alpha +r\alpha_{1})}$$

and *m* = *C* / *A*, user can decrypt F* to get *F* with *m*.

### Correctness analyses

In this subsection, we show that our construction is correct with some appropriate parameters setting.

(1) In the process of search the equation holds, it means that cloud server selects *N*_1_ keywords index from the *Ind* which we denote $\left\{I_{1,O_{1}},I_{1,O_{2}},\ldots I_{1,O_{N_{1}}}\right\}$,where $1\leq O_{1},\ldots ,O_{N_{1}}\leq N$ is matching the search token of the keywords $\left\{w_{j_{1}},w_{j_{2}},\ldots ,w_{j_{N_{1}}}\},(1\leq j_{1},\ldots ,j_{N_{1}}\leq N\right)$ from the user, then computes that

$$\begin{array}{l} \prod_{q=1}^{N_{1}}e(I_{1},\tau_{1,j_{q}})\\ =\prod_{q=1}^{N_{1}}e(g^{\beta },g^{\beta }\cdot H(w_{j_{q}}))\\ =\prod_{q=1}^{N_{1}}e(g^{\beta },I_{1}\cdot H(w_{j_{q}}))\\ =\prod_{\sigma =1}^{N_{1}}e(I_{1},I_{1,O_{\sigma }}) \end{array}$$

a. When *ID* ∉ *R*_*θ*_, compute that

$$\begin{array}{l} \frac{e{\left(I_{0},\prod_{i\in I}(\tau_{1,i}\cdot \tau_{2,0}^{\rho (i)}\right)}^{\mu_{i}})}{e({\prod_{i\in I}\left(I_{2,0}^{\rho (i)}\right)}^{\mu_{i}},g)}\\ =\frac{e(g^{t},g^{\sum_{i\in I}\lambda_{i}\mu_{i}}\cdot \prod_{i\in I}K_{0}^{\xi_{i}\mu_{i}}(\rho (i)))}{e(\prod_{i\in I}K_{0}^{t\xi_{i}\mu_{i}}(\rho (i)),g)}\\ =\frac{e(g^{t},g^{\beta })\cdot e(g,\prod_{i\in I}K_{0}^{\xi_{i}\mu_{i}}(\rho (i)){)}^{t}}{e{\left(\prod_{i\in I}K_{0}^{\xi_{i}\mu_{i}}(\rho (i)),g\right)}^{t}}\\ =e(g^{t},g^{\beta })\\ =e(I_{0},I_{1}) \end{array}$$

b. When *ID* ∈ *R*_*θ*_, compute that

$$\begin{array}{ll} & \frac{e{\left(I_{0},\prod_{i\in I}(\tau_{1,i}\cdot \tau_{2,1}^{\rho (i)}\right)}^{\mu_{i}})}{e({\prod_{i\in I}\left(I_{2,1}^{\rho (i)}\right)}^{\mu_{i}},g)}\\ = & \frac{e(g^{t},g^{\sum_{i\in I}\lambda_{i}\mu_{i}}\cdot \prod_{i\in I}K_{1}^{\xi_{i}\mu_{i}}(\rho (i)))}{e(\prod_{i\in I}K_{1}^{t\xi_{i}\mu_{i}}(\rho (i)),g)}\\ = & \frac{e(g^{t},g^{\beta })\cdot e(g,\prod_{i\in I}K_{1}^{\xi_{i}\mu_{i}}(\rho (i)){)}^{t}}{e{\left(\prod_{i\in I}K_{1}^{\xi_{i}\mu_{i}}(\rho (i)),g\right)}^{t}}\\ = & e(g^{t},g^{\beta })\\ = & e(I_{0},I_{1}) \end{array}$$

(2) The decryption process first calculates

$$\begin{array}{l} \begin{array}{l} K_{i}={\left(h_{1}^{-\frac{x_{i}}{x1}}\cdot h_{i}\right)}^{r}\\ ={\left(g_{}^{-\frac{x_{i}}{x1}\cdot \alpha_{1}}\cdot g^{\alpha_{i}}\right)}^{r}\\ =g^{r\cdot \left(-\frac{x_{i}}{x1}\cdot \alpha_{1}+\alpha_{i}\right)} \end{array}\\ M_{X}=\left(\begin{array}{c} -\frac{x_{2}}{x1}-\frac{x_{3}}{x1}\ldots -\frac{x_{n}}{x1}\\ I_{n-1} \end{array}\right)\\ \left(\begin{array}{cc} \begin{array}{l} -\frac{x_{2}}{x1}\\ -\frac{x_{3}}{x1}\\ \vdots \\ -\frac{x_{n}}{x1} \end{array} & I_{n-1} \end{array}\right)\cdot \left(\begin{array}{l} \alpha_{1}\\ \alpha_{2}\\ \vdots \\ \alpha_{n} \end{array}\right)=\left(\begin{array}{l} -\frac{x_{2}}{x1}\cdot \alpha_{1}+\alpha_{2}\\ -\frac{x_{3}}{x1}\cdot \alpha_{1}+\alpha_{3}\\ \vdots \\ -\frac{x_{n}}{x1}\cdot \alpha_{1}+\alpha_{n} \end{array}\right)=M_{{}_{X}}^{\text{T}}\cdot A\\ K_{X}=\{K_{2},\cdots ,K_{n}\}=g^{r\cdot M_{{}_{X}}^{\text{T}}\cdot A} \end{array}$$

(3) The decryption process calculates:

a. When *ID* ∈ *R*_*θ*_

$$\begin{array}{ll} \varphi & ={\prod_{i\in I}\left(\frac{e(C_{1},D_{1,1}^{\left(i\right)})}{e(C_{2,1}^{\rho (i)},D_{2,1}^{\left(i\right)})}\right)}^{\mu_{i}}\\ & ={\prod_{i\in I}\left(\frac{e(g^{s},g^{\lambda_{i,1}}T_{1}{\left(\rho (i)\right)}^{r_{i,1}})}{e(T_{1}{\left(\rho (i)\right)}^{s},g^{r_{i,1}})}\right)}^{\mu_{i}}\\ & ={\prod_{i\in I}\left(\frac{e(g^{s},g^{\lambda_{i,1}})\cdot e(g^{s},T_{1}{\left(\rho (i)\right)}^{r_{i,1}}))}{e(T_{1}{\left(\rho (i)\right)}^{s},g^{r_{i,1}})}\right)}^{\mu_{i}}\\ & ={\prod_{i\in I}\left(e(g^{s},g^{\lambda_{i,1}})\right)}^{\mu_{i}}\\ & =\prod_{i\in I}e{\left(g,g\right)}^{s\cdot \lambda_{i,1}\cdot \mu_{i}}\\ & =e{\left(g,g\right)}^{s\cdot (\sum_{i\in I}\lambda_{i,1}\cdot \mu_{i})}\\ & =e{\left(g,g\right)}^{s\alpha } \end{array}$$

b. When *ID* ∉ *R*_*θ*_

$$\begin{array}{ll} K_{X} & =\prod_{i=2}^{n}K_{{}_{i}}^{y_{i}}\\ & =\prod_{i=2}^{n}{\left(h_{1}^{-\frac{x_{i}}{x1}}\cdot h_{i}\right)}^{r\cdot y_{i}}\\ & ={\left(h_{1}^{-\left(\frac{x_{2}y_{2}}{x1}+\cdots +\frac{x_{n}y_{n}}{x1}\right)}\cdot \prod_{i=2}^{n}h_{i}{}^{y_{i}}\right)}^{r}\\ & ={\left(h_{1}^{-\left(\frac{x_{2}y_{2}}{x1}+\cdots +\frac{x_{n}y_{n}}{x1}\right)}\cdot \prod_{i=1}^{n}h_{i}{}^{y_{i}}\cdot h_{1}{}^{-y_{1}}\right)}^{r}\\ & ={\left(h_{1}^{-\left(\frac{x_{2}y_{2}}{x1}+\cdots +\frac{x_{n}y_{n}}{x1}\right)}\cdot \prod_{i=1}^{n}h_{i}{}^{y_{i}}\cdot h_{1}{}^{-\frac{y_{1}x_{1}}{x_{1}}}\right)}^{r}\\ & ={\left(h_{1}^{-\left(\frac{x_{1}y_{1}}{x1}+\cdots +\frac{x_{n}y_{n}}{x1}\right)}\cdot \prod_{i=1}^{n}h_{i}{}^{y_{i}}\right)}^{r}\\ & ={\left(h_{1}^{-\frac{<X,Y>}{x1}}\cdot \prod_{i=1}^{n}h_{{}_{i}}^{y_{i}}\right)}^{r} \end{array}$$

$$\begin{array}{ll} \phi & ={\left(\frac{e(K,C_{1})}{e(C_{3},D_{3})}\right)}^{-\frac{x_{1}}{<X,Y>}}\\ & ={\left(\frac{e\left({\left(h_{1}^{-\frac{<X,Y>}{x1}}\cdot \prod_{i=1}^{n}h_{{}_{i}}^{y_{i}}\right)}^{r},g^{s}\right)}{e\left({\left(h_{{}_{1}}^{y_{1}}\cdots h_{{}_{n}}^{y_{n}}\right)}^{s},g^{r}\right)}\right)}^{-\frac{x_{1}}{<X,Y>}}\\ & ={\left(\frac{e\left(\left(h_{1}^{-\frac{<X,Y>}{x1}}\right),g\right)\cdot e\left(\left(\prod_{i=1}^{n}h_{{}_{i}}^{y_{i}}\right),g\right)}{e\left(\left(h_{{}_{1}}^{y_{1}}\cdots h_{{}_{n}}^{y_{n}}\right),g\right)}\right)}^{-\frac{x_{1}}{<X,Y>}\cdot r\cdot s}\\ & ={\left(e\left(\left(h_{1}^{-\frac{<X,Y>}{x1}}\right),g\right)\right)}^{-\frac{x_{1}}{<X,Y>}\cdot r\cdot s}\\ & ={\left(e\left(\left(g_{}^{-\frac{<X,Y>}{x1}\cdot \alpha_{1}}\right),g\right)\right)}^{-\frac{x_{1}}{<X,Y>}\cdot r\cdot s}\\ & =e{\left(g,g\right)}^{rs\alpha_{1}} \end{array}$$

$$\begin{array}{l} \gamma ={\prod_{i\in I}\left(\frac{e(C_{1},D_{1,0}^{\left(i\right)})}{e(C_{2,0}^{\rho (i)},D_{2,0}^{\left(i\right)})}\right)}^{\mu_{i}}\\ \;={\prod_{i\in I}\left(\frac{e(g^{s},g^{\lambda_{i,0}}T_{0}{\left(\rho (i)\right)}^{r_{i,0}})}{e(T_{0}{\left(\rho (i)\right)}^{s},g^{r_{i,0}})}\right)}^{\mu_{i}}\\ \;={\prod_{i\in I}\left(\frac{e(g^{s},g^{\lambda_{i,0}})\cdot e(g^{s},T_{0}{\left(\rho (i)\right)}^{r_{i,0}}))}{e(T_{0}{\left(\rho (i)\right)}^{s},g^{r_{i,0}})}\right)}^{\mu_{i}}\\ \;={\prod_{i\in I}\left(e(g^{s},g^{\lambda_{i,0}})\right)}^{\mu_{i}}\\ \;=\prod_{i\in I}e{\left(g,g\right)}^{s\cdot \lambda_{i,0}\cdot \mu_{i}}\\ \;=e{\left(g,g\right)}^{S\cdot \left(\sum_{i\in I}\lambda_{i,0}\cdot \mu_{i}\right)}\\ \;=e{\left(g,g\right)}^{s\cdot (\alpha +r\alpha_{1})} \end{array}$$

Let *A* = *γ* / *ϕ* = *e*(*g*, *g)*^*sα*^.

## Security analyses

### Selective security model proof

**Theorem1**. If an adversary can break our scheme with advantage *ε* in the selective security model, then we can construct a simulator to solve the Decision q-BDHE problem with advantage $\frac{\varepsilon }{2}$.

*Proof*: This proof bases on [24].

The simulation proceeds as follows. First, the challenger sets

$$Y=(g,g_{}^{s},g_{1}=g^{a},g_{2}=g^{a^{2}},\ldots ,g_{q}=g_{}^{a^{q}},g_{q+2}=g_{{}_{}}^{a^{q+2}},\ldots ,g_{2q}=g_{}^{a^{2q}})$$

Then the challenger flips a fair binary coin *μ*: if *μ* = 0, the challenger sets *Z* = *e*(*g*_1_, *g*_*q*_)^*s*^ if *μ* = 1,then the challenger picks a random element *Z* from *G*_2_.

**Init**. The simulator B runs adversary A. A selects an attribute set ω* and a user revocation list $R_{\theta }^{*}$,where *θ* ∈ ω*, which it wishes to be challenged upon.

**Setup**. The simulator B proceeds as follows:

(1) The simulator B randomly chooses *α*′, *β*, *δ*, ∈ *Z*_*p*_, and then simulator B sets that $e{\left(g,g\right)}^{\alpha }=e(g^{a},g^{a^{q}})\cdot e{\left(g,g\right)}^{\alpha^{\prime }}$,implicitly has that *α* = *α′ + α*^*q+1*^. Then it randomly chooses $\left\{k_{0,i}^{\prime },k_{1,i}^{\prime }\in G_{1}{|}_{i=1,\ldots ,m}\right\}$, and computes

$$K_{0}(x)=\prod_{i=1}^{m}k_{0,i}^{\prime (x^{i})},K_{1}(x)=\prod_{i=1}^{m}k_{1,i}^{\prime (x^{i})}$$

(2) It sets $R_{\theta }^{*}=\{ID_{1},\cdots ,ID_{m}\}$ where *m* ≤ *Q*. For *k* ∈ [1, *m*], simulator B sets $X_{k}=(x_{k,1},\ldots ,x_{k,n})=(1,ID_{k},ID_{k}^{2},\ldots ,ID_{k}^{n-1})$, randomly chooses **b**_*k*_ ∈ *Z*_*p*_ and has that

$$b_{k}^{\text{T}}\cdot M_{X_{k}}=b_{k}^{\text{T}}\cdot \left(\begin{array}{c} -\frac{x_{k,2}}{x_{k,1}}\ldots -\frac{x_{k,n}}{x_{k,1}}\\ I_{n-1} \end{array}\right)=0$$

and $b_{k}={\left(1,\frac{x_{k,2}}{x_{k,1}},\ldots \frac{x_{k,n}}{x_{k,1}}\right)}^{\text{T}}$. The simulator B sets the *n*×*q* matrix **B** = (**b**_1_**|…|b**_*m*_|**0**|…|**0**), for *k* ∈ [1, *m*], it consists by **b**_*k*_, and *q* − *m* columns are **0**. Sets **Z** = (*z*_1_,⋯,*z*_*q*_)^T^ ∈ *Z*^*n*^ and *z*_*i*_ = *a*^*q*+1−*i*^, $g^{z}={\left(g^{a_{q}},\cdots ,g^{a}\right)}^{\text{T}}$ and implicitly has that **A = B·Z + δ** where $\delta \overset{R}{\in }Z_{p}^{n}$. Define **H** = (*h*_1_, *h*_2_,…,*h*_*n*_)^T^ = *g*^**B**·**Z**^·*g*^**δ**^, for *k* ∈ [1, *m*], we have that $M_{X_{k}}^{\text{T}}\cdot B\in {\text{(}Z_{p}\text{)}}^{(n-1)\times q}=0$, so it doesn’t have *z*_*k*_ = *a*^*q*+1−*k*^.

(3) It sets ω*′ = ω* − {*θ*}, randomly chooses two polynomials *f*_0_(*x*) and *f*_1_(*x*) of degree *m* and computes two polynomials as follows:

$$\begin{array}{l} u_{0}(x)=x^{m-\left|\omega^{*}\right|}\prod_{i\in \omega^{*}}\left(x-i\right)\\ u_{1}(x)=x^{m-\left|\omega^{*}-\{\theta \}\right|}\prod_{i\in \omega^{*}-\{\theta \}}\left(x-i\right) \end{array}$$

For *i* ∈ [0, *m*], let *c*_0,*i*_ and *c*_1,*i*_ be the *i*th term of *f*_0_(*x*) and *f*_1_(*x*), *d*_0,*i*_ and *d*_1,*i*_ be the *i*th term of *u*_0_(*x*) and *u*_1_(*x*). B defines $T_{0}(x)=g^{a\cdot u_{0}(x)+f_{0}(x)}$ and $T_{1}(x)=g^{a\cdot u_{1}(x)+f_{1}(x)}$,at the same time, B simulates {*t*_0,*i*_, *t*_1,*i*_}_*i* = 1,…,*m*_ where

$$t_{0,i}={\left(g^{a}\right)}^{d_{0,i}}g^{c_{0,i}},t_{1,i}={\left(g^{a}\right)}^{d_{1,i}}g^{c_{1,i}}$$

Finally, B gives the public parameters

$$pp=<g,e{\left(g,g\right)}^{\alpha },H={\left(h_{1},h_{2},\ldots ,h_{n}\right)}^{\text{T}},g^{\beta },\delta ,K_{0}(x),K_{1}(x)>$$

to A.

**Phase 1**. Let *M* be a *p*×*l* matrix, ω*′ doesn’t satisfy the access structure (*M*, *ρ*). If *ID* ∈ *R*_*θ*_, there is ω*′ = ω* − {*θ*}; otherwise, ω*′ = ω*. The simulator B generates the secret key *sk* as follows.

(1) When *ID* ∉ *R*_*θ*_ (in this case, we have ω*′ = ω*), and ω*′doesn’t satisfy the access structure, B first defines $\pi ={\left(\pi_{1},\cdots ,\pi_{l}\right)}^{\text{T}}\in Z_{p}^{n*}$ where *π*_1_ = 1 We have *M*_*i*_·***π*** = 0 for each *i* when *ρ*(*i*) ∈ ω*. Then the simulator B defines two vectors **η**_0_ = (*r*, *η*_0,2_,…,*η*_0,*l*_)^T^ and **η**_1_ = (0, *η*_1,2_,…,*η*_1,*l*_)^T^, and defines that **u**_0_ = *α*_1_ **η**_0_ + *α***π** and **u**_1_ = **η**_1_ + *α***π**, we can compute the first term of **u**_0_ and **u**_1_ are *α* + *rα*_1_ and *α*.

i. When *ρ*(*i*) ∈ ω*, B computes that

$$g^{\lambda_{i,0}}=g^{M_{i}\cdot \mu_{0}}={\left(g^{\alpha_{1}}\right)}^{M_{i}\cdot \eta_{0}},g^{\lambda_{i,1}}=g^{M_{i}\cdot \eta_{1}}$$

and randomly chooses *r*_*i*,0_, *r*_*i*,1_ ∈ *Z*_*p*_ and computes that

$$D_{1,0}^{\left(i\right)}=g^{\lambda_{i,0}}T_{0}{\left(\rho (i)\right)}^{r_{i,0}},D_{2,0}^{\left(i\right)}=g_{}^{r_{i,0}}$$

$$D_{1,1}^{\left(i\right)}=g^{\lambda_{i,1}}T_{1}{\left(\rho (i)\right)}^{r_{i,1}},D_{2,1}^{\left(i\right)}=g_{}^{r_{i,1}}$$

ii. When *ρ*(*i*) ∉ ω*, B computes that

$$g^{\lambda_{i,0}}=g^{M_{i}\cdot {\text{u}}_{0}}=g^{\alpha_{1}\cdot M_{i}\cdot \eta_{0}+\alpha \cdot M_{i}\cdot \pi },g^{\lambda_{i,1}}=g^{M_{i}\cdot {\text{u}}_{1}}=g^{M_{i}\cdot \eta_{1}+\alpha \cdot M_{i}\cdot \pi }$$

and randomly chooses $r,{\left\{r_{i,0}^{\prime }\right\}}_{i\in [l]},{\left\{r_{i,1}^{\prime }\right\}}_{i\in [l]}\in Z_{p}$, and sets $r_{i,0}=r_{i,0}^{\prime }-\frac{a^{q}}{\mu_{0}(\rho (i))}(M_{i}\cdot \pi )$ and $r_{i,1}=r_{i,1}^{\prime }-\frac{a^{q}}{\mu_{1}(\rho (i))}(M_{i}\cdot \pi )$, then

$$\begin{array}{ll} D_{1,0}^{\left(i\right)} & =g^{\lambda_{i,0}}T_{0}{\left(\rho (i)\right)}^{r_{i,0}}\\ & =g^{\alpha_{1}\cdot M_{i}\cdot \eta_{0}+\alpha \cdot M_{i}\cdot \pi }T_{0}{\left(\rho (i)\right)}^{r_{i,0}^{\prime }}g^{-\frac{a^{q}\cdot f_{0}(\rho (i))\cdot (M_{i}\cdot \pi )}{u_{0}(\rho (i))}} \end{array}$$

$$D_{2,0}^{\left(i\right)}=g_{}^{r_{i,0}}=g^{r_{i,0}^{\prime }-\frac{a^{q}}{\mu_{0}(\rho (i))}(M_{i}\cdot \pi )}$$

$$\begin{array}{ll} D_{1,1}^{\left(i\right)} & =g^{\lambda_{i,1}}T_{1}{\left(\rho (i)\right)}^{r_{i,1}}\\ & =g^{M_{i}\cdot \eta_{1}+\alpha \cdot M_{i}\cdot \pi }T_{1}{\left(\rho (i)\right)}^{r_{i,1}^{\prime }}g^{-\frac{a^{q}\cdot f_{1}(\rho (i))\cdot (M_{i}\cdot \pi )}{u_{1}(\rho (i))}} \end{array}$$

$$D_{2,1}^{\left(i\right)}=g_{}^{r_{i,1}}=g^{r_{i,1}-\frac{a^{q}}{\mu_{1}(\rho (i))}(M_{i}\cdot \pi )}$$

Then B computes that *D*_3_ = *g*^*r*^, $K_{X}={\left\{K_{i}={\left(h_{1}^{-\frac{x_{i}}{x_{1}}}\cdot h_{i}\right)}^{r}\right\}}_{i\in [2,\ldots ,n]}$.

(2) When $ID\in R_{\theta }^{*}$ and sets ${\left\{ID=ID_{k}\right\}}_{k\in [1,m]}$. The simulator B randomly chooses *r*′ ∈ *Z*_*p*_ and sets *r* = *r*′ − *a*^*k*^. Defines **A** = **B** · **Z**+**δ**, the first term of **A** is $\alpha_{1}=\delta_{1}+\sum_{j=1}^{m}a^{q+1-j}$, and computes that

$$\begin{array}{lll} g^{\alpha +r\alpha_{1}} & = & g^{\alpha^{\prime }+a^{q+1}}\cdot {\left(g^{\delta_{1}+\sum_{j=1}^{m}a^{q+1-j}}\right)}^{r^{\prime }-a^{k}}\\ & = & g^{\alpha^{\prime }-\delta_{1}a^{k}}\cdot g^{\alpha_{1}r^{\prime }}\cdot g^{-(\sum_{j=1,j\neq k}^{m}a^{q+1-j+k})} \end{array}$$

randomly chooses ${\left\{\eta_{i}^{}\right\}}_{i\in [2,l]}\in Z_{p}$ and defines **η** = (*α* + *rα*_1_, *η*_2_, …, *η*_*l*_)^T^, and for *i* ∈ [1, *p*], sets *M*_*i*_ = (*x*_*i*,1_, *x*_*i*,2_, …, *x*_*i*,*l*_), then computes

$$g^{\lambda_{i,0}}=g^{M_{i}\cdot \eta }={\left(g^{\alpha +r\alpha_{1}}\right)}^{x_{i,1}}g^{\sum_{j=2}^{l}\eta_{j}\cdot x_{i,j}}$$

randomly chooses *r*_*i*,0_ ∈ *Z*_*p*_, then

$$D_{1,0}^{\left(i\right)}=g^{\lambda_{i,0}}T_{0}{\left(\rho (i)\right)}^{r_{i,0}},D_{2,0}^{\left(i\right)}=g^{r_{i,0}}$$

As ω*′ does not satisfy the access structure, the simulation of $D_{1,1}^{\left(i\right)}$ and $D_{2,1}^{\left(i\right)}$ are the same as the previous case. For {*K*_*i*_}_*i*∈[2,*n*]_, the simulator B can computes $K_{X}=(K_{2},\ldots ,K_{n})=g^{r\cdot M_{X}^{\text{T}}A}$ by $M_{X}^{\text{T}}A=M_{X}^{\text{T}}\cdot B\cdot Z+M_{X}^{\text{T}}\cdot \delta$.

**Challenge**. The adversary A submits two messages *m*_0_ and *m*_1_, B randomly chooses *m*_*b*_ where *b* ∈{0,1} to encrypt. Then computes

$$C=m_{b}\cdot Z\cdot e(g^{s},g^{\alpha^{\prime }}),C_{1}=g^{s}$$

$$C_{2,0}=\{C_{2,0}^{\left(x\right)}|C_{2,0}^{\left(x\right)}=T_{0}{\left(x\right)}^{s}={\left(g^{s}\right)}^{f_{0}(x)},x\in \omega^{*}\}$$

$$C_{2,1}=\{C_{2,1}^{\left(x\right)}|C_{2,1}^{\left(x\right)}=T_{1}{\left(x\right)}^{s}={\left(g^{s}\right)}^{f_{1}(x)},x\in \omega^{*}-\{\theta \}\}$$

Then the simulator B defines **Y** = (*y*_1_, ⋯, *y*_*n*_)^T^ according to the revocation list $R_{\theta }^{*}$ and <**X**_*k*_, **Y** > = 0 for *k* ∈[1,*m*]. And we have that $Y=M_{X_{k}}\cdot \gamma_{1}$ where **γ**_1_ = (*y*_2_, ⋯, *y*_*n*_)^T^, then

$$<Y,B\cdot Z>=Y_{}{}^{\text{T}}B\cdot Z=\sum_{\text{k}=\text{1}}^{m}z_{k}\cdot Y_{}{}^{\text{T}}\cdot b_{k}=0$$

and computes

$$C_{3}={\left(h_{1}^{y_{1}}\ldots h_{n}^{y_{n}}\right)}^{s}={\left(g^{s}\right)}^{<Y,A>}={\left(g^{s}\right)}^{<Y,\delta >}$$

Then B sends the challenge ciphertext ct* = (*C*, *C*_1_, *C*_2,0_, *C*_2,1_, *C*_3_) to the adversary A. If *μ* = 0, then *Z* = e(*g*_1_, *g*_*q*_)^*s*^, the challenge ciphertext ct* is a valid random encryption of message *m*_*b*_. If *μ* = 1, then *Z* is a random element of *G*_2_, and ct*is also random from the adversary’s view, and ct* contains no information of *m*_*b*_.

**Phase2**. Same as **Phase1**.

**Guess**. The adversary A outputs the guess *b*′ of *b*. B outputs *μ* = 0 to guess that *Z* = *e*(*g*_1_, *g*_*q*_)^*s*^ if *b*′ = *b*; otherwise, B outputs *μ* = 1, and it indicates that *Z* is a random element in *G*_2_. And the advantage of simulator B to solve the *q*-BDHE problem is

$$\begin{array}{ll} & \frac{1}{2}\text{Pr}[\mu^{\prime }=\mu |\mu =0]+\frac{1}{2}\text{Pr}[\mu^{\prime }=\mu |\mu =1]-\frac{1}{2}\\ = & \frac{1}{2}(\frac{1}{2}+\varepsilon )+\frac{1}{2}\cdot \frac{1}{2}-\frac{1}{2}\\ = & \frac{\varepsilon }{2} \end{array}$$

### IND-CKA security proof

**Theorem 2**. Suppose there exists a polynomial-time adversary A, which can attack our scheme with advantage *ε* in the IND-CKA model. We can construct a simulator B that can solve the DDH problem in *G*_1_ with probability at lest $\frac{\varepsilon }{4e(M+TN_{1}+\frac{1}{2})}$, where *e* is constant, and we assume the adversary A makes *M* index queries and *T* search token queries(it contains *N*_1_ keywords) in each phase[10].

*Proof*: B is given an instance *g*, *g*^*a*^, *g*^*b*^, *g*^*c*^ of the DDH problem in *G*_1_. In the following parts, we construct the cipher text by setting *δ* = *b*. The simulation proceeds as follows:

**Init**. The adversary A selects a attribute set ω* and a user revocation list $R_{\theta }^{*}$ of *θ* ∈ ω*.B is given an instance *g*, *g*^*a*^, *g*^*b*^, *g*^*c*^ of the DDH problem in *G*_1_. Then B runs the algorithm to generate the public parameter *pp* and sends it to adversary A.

**Phase1. B** maintains a hash list *L* = {*w*_*j*_, *α*_*j*_, *l*_*j*_} and randomly chooses *α*_*j*_ ∈ *Z*_*p*_ for keywords *w*_*j*_ with biased coin flip *l*_*j*_. The list is empty when begins and simulates the hash function as a random oracle. And if the random oracle is queried for a hash of *w*,B searches the hush list *L* if the *w* exists in the list.

1. If *l*_*j*_ = 0,the B gives that $g^{\alpha_{j}}$;
2. If *l*_*j*_ = 1,the algorithm aborts;
3. If the keyword *w* does not exist in the list, the B flips a random coin *l* ∈ {0,1} so that Pr[coin′ = 0] = *σ* and *σ* will be calculated later.
  - If *l* = 0, the B randomly chooses *α* ∈ *Z*_*p*_,and adds < *w*, *α*, 0 > to the hush list;
  - If *l* = 1, the B adds < *w*, ⊥, 1 > to the hush list.
  - The B repeat the above process.

**Keywords index query**. If the adversary A asks the keyword *w*_*j*_ of index information, B searches the hush list *L*. If *l*_*j*_ = 1, B aborts; and if *l*_*j*_ = 0, B randomly chooses *t* ∈ *Z*_*p*_, let $H(w_{j})=g^{\alpha_{j}}$ and generates that

$$I_{0}=g^{t}$$

$$I_{1,j}=g^{\beta }H{\left(w_{j}\right)}^{\delta }=g^{\beta }{\left(g^{b}\right)}^{\alpha_{j}}$$

$$I_{2,0}={\left\{I_{2,0}^{\left(x\right)}=K_{0}^{t}(x)\right\}}_{x\in \omega^{*}},I_{2,1}={\left\{I_{2,1}^{\left(x\right)}=K_{1}^{t}(x)\right\}}_{x\in \omega^{*}-\theta }$$

**Search token query**. If the adversary A asks the keyword $w_{j_{q}}$ of searching token with the access structure (*M*, *ρ*), Let *M* be a *p*×*l* matrix, ω*′doesn’t satisfy the access structure (*M*, *ρ*). If $ID\in R_{\theta }^{*}$, there is ω*′ = ω* − {*θ*}; otherwise, ω*′ = ω*.B searches the hush list *L*. If $l_{j_{q}}=1$,B aborts; and if $l_{j_{q}}=0$,let $H(w_{j_{q}})=g^{\alpha_{j}}$. For *i* = 1 to *l*, randomly choose *ξ*_*i*_ ∈ *Z*_*p*_ and B generates that

$$\begin{array}{lll} \tau_{1}^{*} & = & {\left\{\tau_{1,i,j_{q}}=g^{\lambda_{i}}H{\left(w_{j_{q}}\right)}^{\delta }\right\}}_{i\in [1,l],q\in [1,N_{1}],j_{q}\in [1,N]}\\ \tau_{2,0} & = & {\left\{\tau_{2,0}^{\rho (i)}=K_{0}^{\xi_{i}}(\rho (i))\right\}}_{i\in [1,l]}\\ \tau_{2,1} & = & {\left\{\tau_{2,1}^{\rho (i)}=K_{1}^{\xi_{i}}(\rho (i))\right\}}_{i\in [1,l]} \end{array}$$

**Challenge**. The adversary A outputs two keywords $w_{0}^{*}$ and $w_{1}^{*}$,B randomly chooses *b* ∈ {0,1} and searches the hush list *L* that $<w_{b}^{*},\alpha ,l>$. If *l* = 0,B aborts; if *l* = 1, let $H(w_{b}^{*})=g^{a}$ and computes

$$I_{0}=g^{t},I_{1}=g^{\beta }g^{c}$$

$$I_{2,0}={\left\{I_{2,0}^{\left(x\right)}=K_{0}^{t}(x)\right\}}_{x\in \omega^{*}},I_{2,1}={\left\{I_{2,1}^{\left(x\right)}=K_{1}^{t}(x)\right\}}_{x\in \omega^{*}-\theta }$$

**Phase2**. Same as **Phase1**.

**Guess**. The adversary A outputs the guess *b*′ of *b*, B outputs *g*^*c*^ = *g*^*ab*^ if *b*′ = *b*; otherwise *g*^*c*^ is a random group element in *G*_1_.

**Correctness Analyses**. In the above simulation scheme, if the adversary A has the advantage of attack our scheme, and then it will be given the keyword *w*_*j*_ of hush value is *H*(*w*_*j*_) = *g*^*a*^ rather than the random value *H*(*w*_*j*_) = *g*^*aj*^. Then it can compute that *I*_1_ = *g*^*β*^*H(w)*^*δ*^ = *g*^*β*^(*g*^*b*^)^*a*^, that is *I*_1_ = *g*^*β*^*g*^*c*^ = *g*^*β*^*g*^*ab*^, and B computes that *g*^*c*^ = *g*^*ab*^ which means it solves the DDH problem.

**Probability Analyses**. Suppose that the adversary A makes *M* index queries and *T* search token queries in each phase, and the probability that B will not be terminated in two query phases 1 and 2 is $\sigma^{2(M+TN_{1})}$, so the probability that it will not terminated during the challenge step is 1 − *σ*, so that results in an overall probability that B does not abort is $\sigma^{2(M+TN_{1})}\cdot (1-\sigma )$. And, through the computes that the maximum is $\sigma =1-\frac{1}{2(M+TN_{1})+1}$, so the maximum probability is $\frac{1}{2e(M+TN_{1}+\frac{1}{2})}$. Thus, if our scheme can be attacked by the adversary A with the advantage *ε*, and the B can resolve the DDH problem with advantage $\frac{\varepsilon }{4e(M+TN_{1}+\frac{1}{2})}$.

## Performance analyses

In this section, we give some performance analysis in our scheme. The hardware runtime environment is Intel Core i5-3470 CPU @ 3.20GHz, and RAM is 4.00GB. The software runtime environment is JDK 1.7.5, JPBC 2.0.0 and MyEclipse10.

Our scheme is compared with the schemes of [21, 24, 26, 27, 28] in Table 1.

**Table 1:** Performance analyses.

Scheme	Fine-grained	Attribute revocation	Keyword search	Do not update cipher-text when attribute revocation
[26]	×	×	×	×
[21]	×	×	√	×
[24]	√	√	×	√
[27]	×	×	×	_
[28]	×	×	×	_
Our scheme	√	√	√	√

Our scheme is also compared with the schemes of [26, 27, 28] in Table 2.

**Table 2:** Calculation analyses.

Scheme	KeyGen	Encryption	Pairings in Decryption
[26]	(2 + 2*l*)*ex*	(3 + | *S* |)*ex*	2 + 2| *I* |
[27]	*3lex*	(2 + | *S* |)*ex*	1 + 3| *I* |
[28]	2*lex*	(6 + | *S* |)*ex*	1 + 2| *I* |
Our scheme	(2 + 4*l*)*ex*	(3 + 2 | *S* |)*ex*	1 + 2| *I* |

| *S* |: The size of the attributes set of a decryption key.

*l*: The number of rows of the matrix in access policy(*M*,*ρ*).

*ex*: An exponentiation operation.

| *I* |: The number of attributes for a decryption key to satisfy a cipher-text policy.

We can see from Table 2, our scheme has a large amount of computation in the KenGen and Encryption generation, because our scheme doesn’t need to update the cipher-text and secret key when attributes revocation. However, the schemes of [26], [27] and [28] don’t achieve the function of attribute revocation.

**Fig 2:**
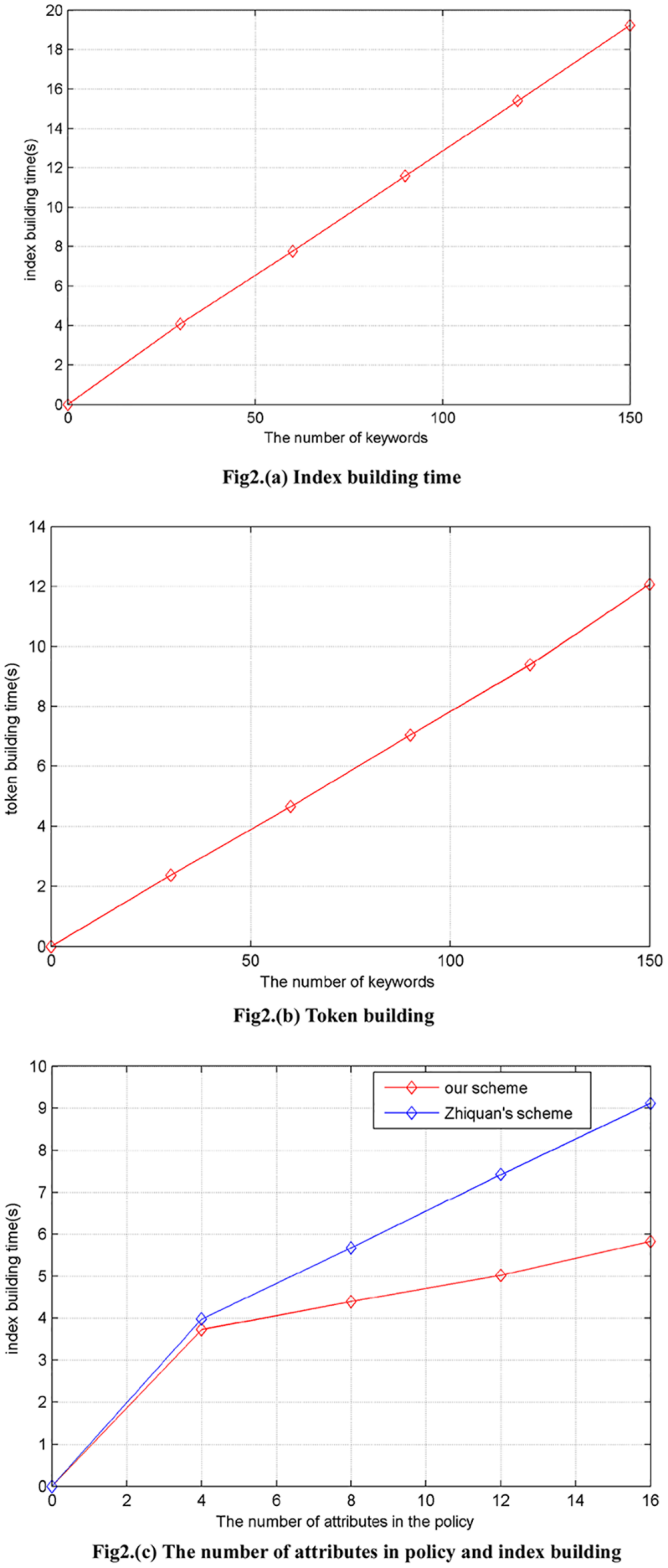
(a) Index building time (b) Token building time (c) The number of attributes in policy and index building time

As is shown in the Fig 2, we suppose that there are 16 attributes in the policy and provide the relational graphs of keywords index building time as is shown in Fig 2(a) and search token building time as is shown in Fig 2(b). From the Fig 2(a) and 2(b), we can see that the time cost is nearly linear with the index building and token building. In the Fig 2(c), we give the relational graph of the number of attributes in the policy and time cost. As is shown in the Fig 2(c), we can find that the effect of the increase of the attributes on the time is not particularly evident in our scheme which takes less time than Zhiquan’s[29].

## Conclusions

In our scheme, we add the keyword search based on the attribute revocation, the search tokens generated by the attribute authority and the user. The cloud server match is divided into two cases: the user is in the revocation list and not in the revocation list, and the cloud server uses the different test according to the different case. It will return the cipher text when the attribute set meets the access structure and the search keywords exist, and the user can decrypt correctly. This scheme supports multiple keywords search at the same time which makes more flexible in the practical application.

## Supporting information

S1 Appendix (RAR)
